# Submassive Pulmonary Embolism in Mild COVID-19 Without Lung Infiltrates

**DOI:** 10.7759/cureus.13978

**Published:** 2021-03-18

**Authors:** Gaurav Manek, Manasvi Gupta, Soontharee Congrete, Debapriya Datta

**Affiliations:** 1 Department of Internal Medicine, University of Connecticut Health, Farmington, USA; 2 Department of Pulmonary, Critical Care and Sleep Medicine, University of Connecticut Health, Farmington, USA

**Keywords:** covid-19, mild disease, pulmonary embolism, corona virus, lung infiltrates, hypercoagulable state, sars cov-2

## Abstract

A 66-year-old man who had been diagnosed with mild coronavirus 2019 (COVID-19) infection nine days prior presented to the emergency room with acute-onset chest pain and shortness of breath. Chest CT angiogram (CTA) revealed pulmonary emboli (PE) in the right and left pulmonary arteries with right heart strain; lung parenchyma showed no infiltrates. Although severe COVID-19 infection is associated with thrombotic complications, data regarding the occurrence of PE in mild cases of COVID-19 is scarce. However, even mild cases of COVID-19 are reported to have revealed lung infiltrates, particularly ground-glass opacities, on imaging. The possibility of the lungs being the primary source of COVID-19-associated coagulopathy has been raised. We report an uncommon case of submassive PE occurring in mild COVID-19, without any associated lung infiltrates. This case indicates that mild COVID-19, without significant lung parenchymal involvement, can also cause a hypercoagulable state, resulting in venous thromboembolism (VTE).

## Introduction

Venous thromboembolism (VTE) has been reported to occur in severe cases of coronavirus disease 2019 (COVID-19) due to the hypercoagulable state caused by the dysregulated immune response resulting from the virus [1,2,3]. COVID-19 causes pneumonic infiltrates consisting of multi-lobar peripheral ground-glass opacities in mild-to-moderate and severe disease [4]. The possibility of the lungs being the primary source of COVID-19-associated coagulopathy has been raised [5]. However, data regarding deep vein thrombosis (DVT) and pulmonary embolism (PE) occurring with mild COVID-19 are scarce. In this report, we present a case of mild COVID-19, without lung infiltrates, complicated by submassive PE and lower extremity DVT.

## Case presentation

**Figure 1:**
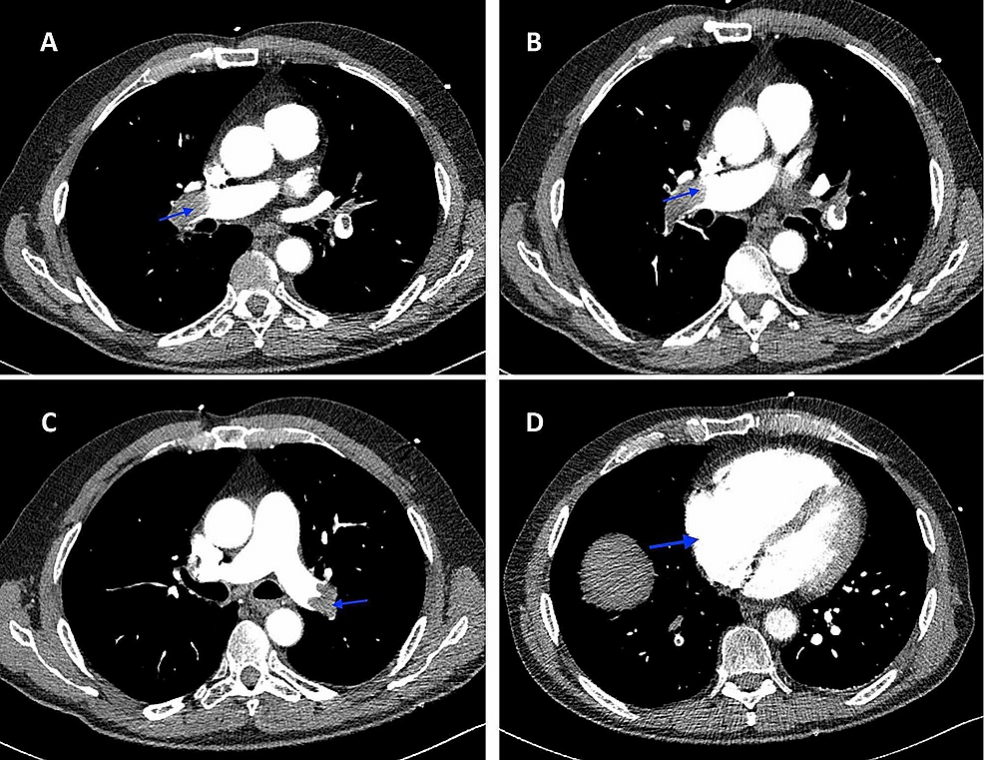
Chest CTA revealing large filling defects in the right and left main pulmonary arteries (A, B, C) with right heart strain (D) CTA: computed tomography angiography

**Figure 2:**
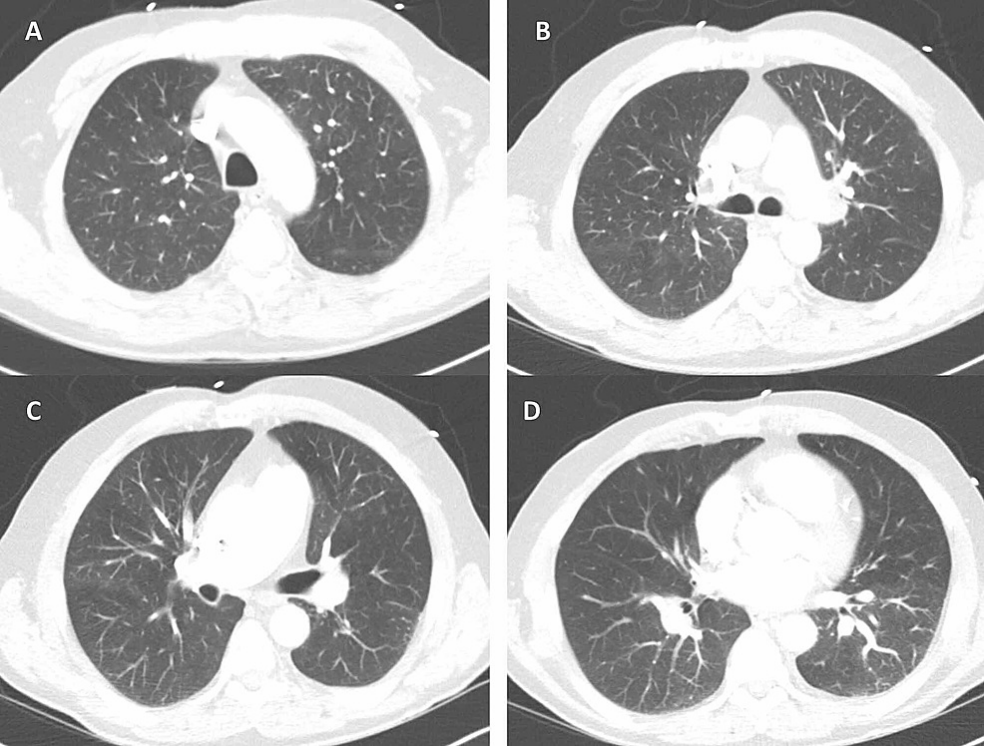
Chest CTA with no lung infiltrates present CTA: computed tomography angiography

**Figure 3:**
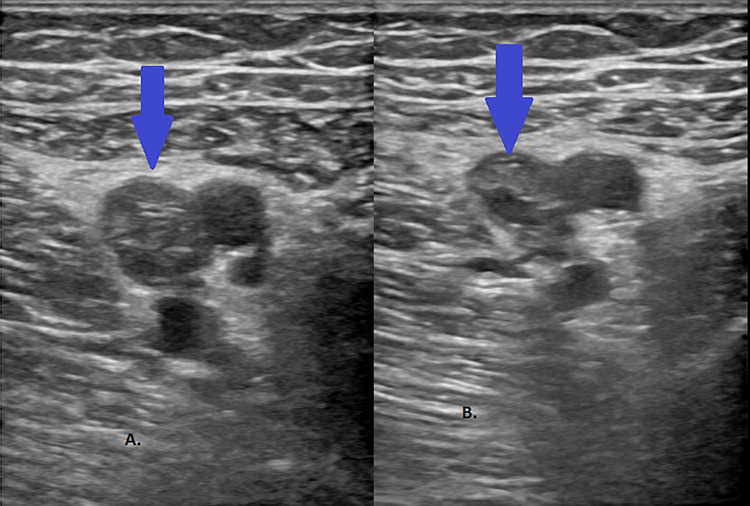
Venous Doppler ultrasound of the left lower extremity revealing DVT of the left proximal femoral vein (A) and a non-compressible left proximal femoral vein (B) DVT: deep vein thrombosis

A 66-year-old male with a past medical history of hypertension presented to the emergency room with acute-onset chest pain and shortness of breath. He had been diagnosed with COVID-19 nine days prior after developing fever and cough, but those symptoms had since resolved. The patient complained of fatigue and reduced oral intake following his diagnosis of COVID-19. He had no family history of VTE, no recent travel, no recent surgery or immobilization, and was up to date with his cancer screening. On examination, the patient was in no distress. Vital signs on admission were as follows - temperature: 98 ℉; heart rate: 97 beats/minute; blood pressure: 92/64 mmHg; respiratory rate: 20 breaths/minute; and oxygen (O_2_) saturation: 87% on room air. The physical examination was normal except for elevated jugular venous pressure. Investigations including complete blood count, basic metabolic panel, lactate dehydrogenase, ferritin, and lactic acid levels were normal. D-dimer was elevated at 8,583 ng/mL, C-reactive protein was 62.4 mg/L, and troponin was 0.44 ng/mL. Chest CT angiogram (CTA) revealed large filling defects in the right and left main pulmonary arteries with right heart strain (Figure 1). No lung infiltrates were present (Figure 2). Ultrasound of lower extremities revealed DVT of the left proximal femoral vein (Figure 3).

The patient was administered 500 ml of normal saline with improvement in blood pressure to 126/74 mmHg. O_2_ saturation improved to 96% on O_2_ at 3 L/minute via nasal cannula. Anticoagulation with intravenous heparin was started. Due to concern for additional PE burden from his extensive DVT, an inferior vena cava filter was placed. Over the next 24 hours, his symptoms and hypoxia improved, and he was subsequently discharged on apixaban.

## Discussion

Thrombotic events are known complications of COVID-19 infection. Studies have shown that up to 25-31% of critically ill patients with COVID-19 suffer from VTE [1,2,3]. COVID-19 is hypothesized to cause endothelial cell inflammation and dysfunction, abnormal flow dynamics, and platelet activation, which together with other viral factors result in factor XI activation and thrombin generation, leading to VTE [5]. Questions have been raised as to whether the lungs may be the primary source of COVID-19-associated coagulopathy.

PE has been reported to occur mostly with severe COVID-19, with associated lung parenchymal involvement on imaging including multilobar peripheral ground-glass opacities and consolidation [5,6]. The occurrence of VTE in patients with COVID-19 has been linked to the severity of the disease and is usually associated with the occurrence of COVID-19 pneumonia. Increased incidence of PE has been reported in patients with severe disease requiring ICU admission and is more likely to occur in critically ill COVID-19 patients requiring mechanical ventilation [6,7]. Central PE has been reported to occur in 13%, segmental PE in 51%, lobar PE in 31%, and sub-segmental PE in 5.5% of the cases. Right heart strain on CT was reported in 11% of the cases [8].

The correlation between thrombotic complications and elevated levels of D-dimer in COVID-19 patients has also been reported [1,9]. One study reported the occurrence of PE in mild COVID-19; however, the presence or absence of associated parenchymal involvement was not mentioned [8]. Another case series reported late acute PE in patients with mild COVID-19; however, the patients in the case series were noted to have lung infiltrates [9]. The occurrence of PE even in mild COVID-19 has significant implications since the initiation of anticoagulation presumptively in patients with elevated D-dimers has been shown to improve mortality rates [10,11,12].

To the best of our knowledge, this was an unusual case of submassive PE in mild COVID-19 infection, without associated pneumonia. This case highlights the fact that life-threatening VTE may develop in patients with mild COVID-19 without lung parenchymal involvement.

## Conclusions

This case highlights the fact that mild COVID-19 without significant lung parenchymal involvement can also cause a hypercoagulable state and result in VTE. It raises the question as to whether inflammatory markers such as D-dimer should be checked even in mild COVID-19, without radiographic lung involvement, so that prompt diagnosis and treatment of VTE can be instituted. Early recognition of VTE can lead to prompt initiation of anticoagulant therapy, which will, in turn, contribute to improved mortality rates in patients with COVID-19.

## References

[1] Cui S, Chen S, Li X, Liu S, Wang F (2020). Prevalence of venous thromboembolism in patients with severe novel coronavirus pneumonia. J Thromb Haemost.

[2] Klok FA, Kruip MJHA, van der Meer NJM (2020). Incidence of thrombotic complications in critically ill ICU patients with COVID-19. Thromb Res.

[3] Helms J, Tacquard C, Severac F (2020). High risk of thrombosis in patients with severe SARS-CoV-2 infection: a multicenter prospective cohort study. Intensive Care Med.

[4] Becker RC (2020). COVID-19 update: Covid-19-associated coagulopathy. J Thromb Thrombolysis.

[5] Simpson S, Kay FU, Abbara S (2020). Radiological Society of North America Expert Consensus Statement on Reporting Chest CT Findings Related to COVID-19. Endorsed by the Society of Thoracic Radiology, the American College of Radiology, and RSNA.. Radiol Cardiothorac Imaging.

[6] Grillet F, Behr J, Calame P, Aubry S, Delabrousse E (2020). Acute pulmonary embolism associated with COVID-19 pneumonia detected with pulmonary CT angiography. Radiology.

[7] Middeldorp S, Coppens M, van Haaps TF (2020). Incidence of venous thromboembolism in hospitalized patients with COVID-19. J Thromb Haemost.

[8] Poyiadji N, Cormier P, Patel PY (2020). Acute pulmonary embolism and COVID-19. Radiology.

[9] Vechi HT, Maia LR, Alves MDM (2020). Late acute pulmonary embolism after mild coronavirus disease 2019 (COVID-19): a case series. Rev Inst Med Trop Sao Paulo.

[10] Bikdeli B, Madhavan MV, Jimenez D (2020). COVID-19 and thrombotic or thromboembolic disease: implications for prevention, antithrombotic therapy, and follow-up: JACC State-of-the-Art Review. J Am Coll Cardiol.

[11] Tang N, Bai H, Chen X, Gong J, Li D, Sun Z (2020). Anticoagulant treatment is associated with decreased mortality in severe coronavirus disease 2019 patients with coagulopathy. J Thromb Haemost.

[12] Connors JM, Levy JH (2020). COVID-19 and its implications for thrombosis and anticoagulation. Blood.

